# Infective Endocarditis With a Giant Vegetation on the Tricuspid Valve With a Congenital Ventricular Septal Defect in a 63-Year-Old Patient

**DOI:** 10.1016/j.atssr.2025.06.019

**Published:** 2025-07-22

**Authors:** Hande İştar, Buğra Harmandar

**Affiliations:** 1 Department of Cardiovascular Surgery, Muğla Sıtkı Koçman University Medical Faculty, Muğla, Turkey

## Abstract

Infective endocarditis is an infection of the endocardial tissue of the heart, primarily affecting the cardiac valves. Of various causes, untreated or undiagnosed congenital heart defects are known contributors. This report presents the case of a 63-year-old man with infective endocarditis of the tricuspid valve associated with an uncorrected ventricular septal defect. We describe our elderly patient who underwent successful surgical repair, including tricuspid valve reconstruction and closure of the ventricular septal defect, after a long life uncomplicated by severe pulmonary hypertension.

The incidence of infective endocarditis (IE) is 3 to 10 cases per 100,000 individuals per year.^1^ IE typically affects the left-sided cardiac valves. However, isolated tricuspid valve IE occurs in 2.5% to 3.1% of all patients with IE.^2^ Right-sided valve involvement is often associated with intravenous drug use, tattoos, intracardiac devices, VSDs, and central venous or dialysis catheters.^1^^,^^3^ Of children with IE, 34% have cyanotic cardiac disease and 15% have VSD.^4^ This case involves an elderly patient described to date with tricuspid valve IE associated with an uncorrected perimembranous VSD.

A 63-year-old man was hospitalized with fever, tachycardia, and dyspnea. Physical examination revealed bilateral fine inspiratory crepitations and a pansystolic murmur at the left sternal edge, radiating to the right. He had no implantable devices, catheters, or history of recent dental procedures. Transthoracic and transesophageal echocardiography revealed a 3 × 2-cm vegetation attached to the anterior cusp of the tricuspid valve, dilated right-sided chambers, normal left ventricular function, and an incidentally diagnosed perimembranous VSD (Figure 1A, 1B).

**Figure 1:**
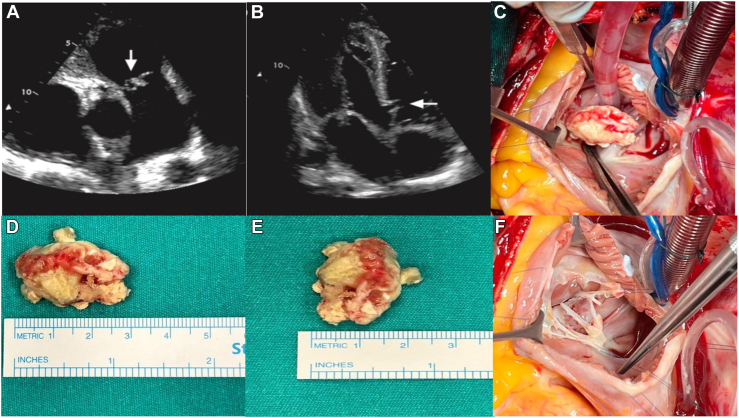
Preoperative and intraoperative findings of tricuspid valve endocarditis and ventricular septal defect repair. (A) Preoperative echocardiogram showing vegetation on the tricuspid valve (arrow). (B) Preoperative echocardiogram showing the ventricular septal defect (arrow). (C) Intraoperative view of the vegetation on the tricuspid valve. (D, E) Vegetation after surgical resection. (F) Tricuspid anterior cusp after resection of the vegetation, showing ruptured chordae.

Trans-VSD shunt was calculated to be 1.8. Coronary angiography showed no significant coronary lesions. Because of the friability of the vegetation on the tricuspid valve, we could not measure the reversibility by a catheter. Chest computed tomography revealed no pulmonary septic emboli.

Preoperative laboratory tests revealed a hematocrit of 26.1%, platelet count of 90,000/μL, and C-reactive protein level of 74 mg/L. Blood cultures identified *Enterococcus faecalis*, and IE was diagnosed by the modified Duke criteria. After 2 weeks of vancomycin and ciprofloxacin therapy, surgery was indicated because of the increasing size of the vegetation.

Surgery was performed under cardiopulmonary bypass by bicaval and aortic cannulation. Under cardioplegic cardiac arrest, a right atriotomy was conducted to explore the tricuspid valve. A 20 × 30-mm vegetation was found on the anterior cusp (Figure 1C-1E). Ruptured secondary chordae were also identified (Figure 1F). All ruptured chordae and the vegetation along with a portion of the anterior cusp were excised (Figure 2A).

**Figure 2:**
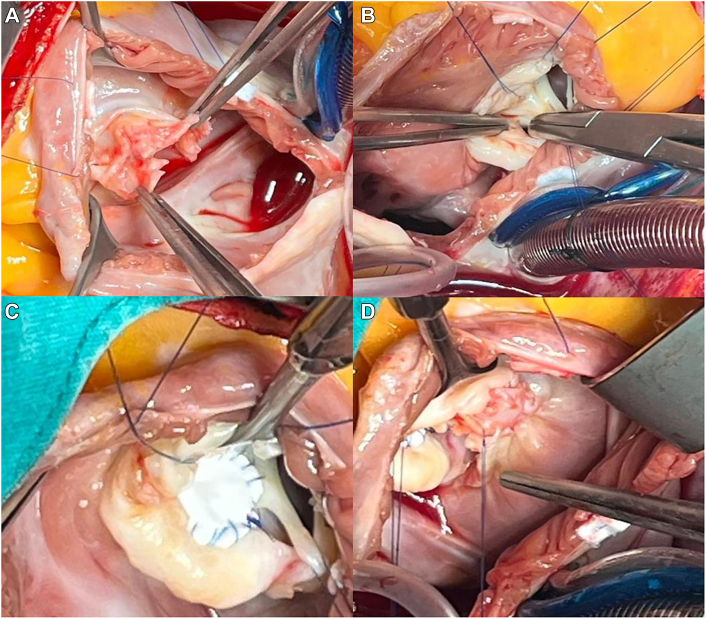
Intraoperative and postoperative findings of tricuspid valve endocarditis and ventricular septal defect (VSD) repair. (A) Defect in the tricuspid anterior leaflet. (B) Intraoperative view of the VSD, demonstrated with a right-angle instrument. (C) Repair of the VSD with a polytetrafluoroethylene graft. (D) Tricuspid anterior leaflet after repair with an autologous pericardial patch.

An 8-mm perimembranous VSD under the septal leaflet pouch (Figure 2B) was closed through the septal leaflet with a 0.6-mm polytetrafluoroethylene graft 12 × 10 mm in size by continuous suturing technique (Figure 2C). The defect in the anterior leaflet was repaired with a glutaraldehyde-treated autologous pericardial patch (Figure 2D). The primary chordae were reattached to the edge of the pericardial patch. A saline test revealed minimal neo–tricuspid valve insufficiency. There was no need of an additional repair with an artificial neochord. Even though the tricuspid annulus was enlarged, it was not excessive; to avoid any conduction system damage for our elderly patient, we preferred not to add any reducing annuloplasty procedure. A probable permanent pacemaker and its leads might cause secondary IE. No operative complications or rhythm disturbances occurred.

Vancomycin and meropenem were administered postoperatively. The patient was extubated on the first postoperative day, and 25 mg of sildenafil 3 times daily was started after extubation. However, atrial fibrillation developed on postoperative day 3. The patient received low-dose inotropic support and was discharged in good condition on postoperative day 10, with sildenafil and acetylsalicylic acid prescribed. Postoperative echocardiography revealed minimal tricuspid valve insufficiency and no residual VSD. Diuretic therapy and acetylsalicylic acid were continued after discharge.

## Comment

Even though congenital heart diseases are rare in the adult population, survival rates for these patients are improving, with adult survival rates reaching >85%.^4^ IE can develop secondary to invasive dental or otolaryngologic procedures, cardiac surgery, pneumonia, trauma, central venous catheters, intracardiac devices, tattoos, or piercings.^3^, ^4^, ^5^, ^6^ Cahill and associates^3^ reported that tetralogy of Fallot and VSD are the most common congenital heart defects in IE, occurring in 22.8% and 19.6% of cases, respectively, across all age groups. Conversely, Lee and coworkers^6^ found that VSD and associated atrial septal defect have a prevalence of 37.5%, whereas tetralogy of Fallot, with or without pulmonary atresia, accounts for 18.1% of cases. Lee and coworkers^6^ also demonstrated that IE is 11 to 15 times more prevalent in individuals with congenital heart defects than in the general population. The most common causative microorganisms in IE affecting native valves are *Streptococcus viridans* and staphylococci, with enterococci accounting for 4% of cases.^3^ The leading causes of death in patients with IE are cardiac failure, cerebral embolization, antimicrobial-resistant infection, pneumonia, and renal failure.

For patients with congenital heart diseases that have remained unknown, IE may develop in very old age owing to turbulent flow on the septal defects. In the literature, Kretzer and coworkers^7^ described a case of IE in a 65-year-old man with Gerbode VSD. In their study, VSD closure and aortic valve replacement were performed because of severe aortic stenosis and a bicuspid aortic valve.

In our 63-year-old patient, the pouch formed by the tricuspid septal leaflet probably restricted the jet flow through the VSD. The combination of the patient’s advanced age and jet flow may have contributed to the rupture of the anterior leaflet chordae. Despite these challenges, we successfully repaired the tricuspid valve with autologous pericardium instead of valve replacement. It should be considered that even today, these cases exist despite a wide spectrum of antibiotics and several types of diagnostic tests. In case of undiagnosed congenital heart diseases in patients of older age, IE might be seen. It is important that medical professionals be alert for IE in case of fever resistant to medical treatment. We believe that elderly patients should not be considered a contraindication to surgery for congenital heart disease complicated with IE.
